# Predictive Modeling of Tourist Satisfaction Based on Service Marketing Mix Elements Using Machine Learning Techniques

**DOI:** 10.1155/tswj/6666970

**Published:** 2026-01-08

**Authors:** Md. Nazmul Hoque, Sumiya Nur Jannat, Yasin Arafat, Md. Mamun Miah

**Affiliations:** ^1^ Department of Marketing, Comilla University, Comilla, Bangladesh, cou.ac.bd; ^2^ Department of Statistics, Noakhali Science and Technology University, Noakhali, Bangladesh, nstu.edu.bd

**Keywords:** Cox′s Bazar, feature importance, machine learning, multicollinearity, service marketing mix, tourist satisfaction, XGBoost

## Abstract

This study examines the impact of the service marketing mix on tourist satisfaction and loyalty, focusing on Cox′s Bazar, Bangladesh. Utilizing data collected from 500 respondents and analyzed through advanced statistical and machine learning techniques, the study provides key insights into the relationships between marketing mix elements and tourist satisfaction. The reliability of the constructs was assessed using Cronbach′s alpha, all of which exceeded the acceptable threshold of 0.70, indicating strong internal consistency. Multicollinearity issues among predictors were resolved by aggregating closely related variables, reducing the variance inflation factor (VIF) to below 1.05. Principal component analysis (PCA) demonstrated that the first two components accounted for 97.88% of the variance, emphasizing the compactness of the data. Predictive modeling revealed that XGBoost outperformed other models with the lowest mean squared error (MSE = 0.10), root mean squared error (RMSE = 0.33), and mean absolute error (MAE = 0.25), alongside the highest *R*‐squared value of 0.74. Feature importance analysis highlighted that the combined variable price_place_aggregated contributed most significantly (68.20%) to the model′s predictions, followed by promotion and process. Cross‐validation confirmed the robustness of the XGBoost model, with a cross‐validated MSE of 0.1273 ± 0.0170. The findings underscore the critical role of pricing strategies and location in enhancing tourist satisfaction and loyalty. This research validates the stability and reliability of the model by integrating sensitivity analysis and learning curve evaluations. These findings offer pragmatic recommendations for policymakers and tourism stakeholders in Cox′s Bazar to enhance their marketing strategies and enhance the entire tourist experience.

## 1. Introduction

Tourism is a dynamic and continually expanding sector that greatly contributes to the economic advancement of numerous countries, including Bangladesh. The industry has bright growth and future possibilities in Bangladesh. As global competition intensifies, understanding and enhancing tourist satisfaction has become a critical priority for destination managers and service providers. Tourist satisfaction is crucial for the success of the tourism sector, as it indicates the profitability of the business and the potential for financial gain [1]. Tourist satisfaction not only influences repeat visits and positive word of mouth but also plays a vital role in sustaining a destination′s long‐term growth [2, 3].

Bangladesh is the eighth‐most populous country in the world with a population of 173,562,364 in an area of 148,460 km^2^ [4]. Bangladesh′s tourism industry, a significant driver of economic prosperity and social progress, faces threats due to its strategic location and its resources [5]. Bangladesh faces challenges in tourism due to inadequate infrastructure, inadequate facilities, scarcity of skilled tour service providers, transportation and lodging facilities, and poor promotional programs [6]. In Bangladesh, there are lots of tourist attractions for international tourists like sunny beaches, historical attractions, hillocks, Sundarbans, lives, cultural diversity, tribal culture and architecture, cultural heritage, and colorful festivals [7]. Travel and tourism are one of the largest service industries in Bangladesh and also in the world [8]. In recent years, tourism has increased in developing countries like Bangladesh. Bangladesh offers numerous international tourist attractions including sunny beaches, historical sites, hillocks, Sundarbans, wildlife, cultural diversity, tribal architecture, heritage, and colorful festivals [7].

Chittagong boasts numerous sea beaches, including Cox′s Bazar, the world′s longest sandy beach, located 120 km away [9]. Cox′s Bazar Beach, one of the world′s longest beaches, is experiencing significant growth in the tourism industry of the country [10]. Bangladesh is the fourth largest and fastest growing tourism industry in the world in terms of employment generation and contribution to national GDP [11, 12]. The attitude of the tourists depends on several factors (product, price, place, promotion, people, process, and physical evidence) related to the destinations [13]. Several studies have been conducted previously to measure the impact of services marketing mix on customer satisfaction in various sectors including hotels, restaurants, tourism, cooperatives, and banks [14, 15]. Tourists were mostly satisfied with natural beauty and relaxation facilities but dissatisfied with the lack of health/emergency services, transportation, tour guides, information services, and nightlife [16].

The application of advanced data‐driven methodologies in tourism research has opened new avenues for understanding and enhancing tourist satisfaction. Machine learning (ML) techniques, in particular, provide powerful tools for predictive modeling by uncovering complex relationships within large datasets and offering actionable insights [17]. Numerous studies across various fields have demonstrated the effectiveness of these methods in finding important factors impacting satisfaction and loyalty [18]. For a place like Cox′s Bazar, which is a cornerstone of Bangladesh′s tourism economy, ML may significantly enhance decision‐making by identifying key indicators of satisfaction and delivering solid recommendations to stakeholders.

This study employs ML approaches to assess the predictive relationships between the service marketing mix elements (product, price, place, promotion, people, process, and physical evidence) and tourist satisfaction in Cox′s Bazar. This study attempts to fill the gap between classic marketing theories and new computational methodologies by combining rigorous statistical analysis with ML capabilities. These results are anticipated to provide strategic insights to Bangladeshi destination managers and policymakers, improving long‐term tourism growth and boosting the worldwide competitiveness of Cox′s Bazar.

## 2. Literature Review

The service marketing mix (7Ps)—product, price, place, promotion, people, process, and physical evidence—has been used as a reference to assess the quality of services within an economy such as in the case of tourism. Past studies indicate that all the 7P elements have a major impact on the satisfaction of the tourists, with the quality of products and people being of great importance to customer retention especially in hotel services [19, 20].

Bangladesh′s tourism industry is rapidly expanding, fueled by popular destinations like Cox′s Bazar. However, challenges such as infrastructure limitations, insufficient promotional efforts, and disparities in service quality hinder the enhancement of tourist satisfaction [21]. Several studies have analyzed the individual components of the marketing mix. The uniqueness and perceived quality of tourist attractions significantly influence satisfaction, emphasizing that tourists prioritize destinations that offer natural beauty, cultural heritage, and high‐quality services [7]. Pricing and place are crucial factors for tourist satisfaction. Previous studies indicate that tourists prefer affordable destinations with convenient access, highlighting the importance of cost‐effective strategies [22, 23]. Digital marketing, social media, and targeted campaigns have proven effective in enhancing tourist engagement [24].

ML has revolutionized the prediction of tourist satisfaction by enabling the analysis of large datasets with complex relationships. Various studies have utilized ML techniques to improve prediction accuracy [25]. XGBoost (extreme gradient boosting) has been widely applied in tourism studies, with previous research demonstrating its superiority in predicting tourist satisfaction metrics compared to traditional models. The XGBoost ML model outperformed support vector machine (SVM) and random forest, achieving the highest accuracy while predicting customer satisfaction [17]. Previous studies reveal the XGBoost method for forecasting various factors associated with tourist attractions and evaluate its performance against other existing methods using metrics such as MAPE, MAE (mean absolute error), RMSE (root mean squared error), and *R* ^2^ [26]. The linear regression model performed the best in predicting both domestic and international visitors to the Moche Route tourist attractions in Peru [17].

In China, studies have shown that the impact of marketing mix elements on tourist satisfaction varies, with all elements except price demonstrating a significant positive relationship with satisfaction [27]. Furthermore, the stage of tourism development, state of infrastructure, and available facilities significantly influence satisfaction levels [28]. The global growth of tourism in recent decades presents significant opportunities for addressing economic and social challenges, particularly in underdeveloped regions rich in natural, cultural, and historical resources [29].

Research on the use of ML techniques to predict tourist preferences in Bangladesh has found that methods like linear SVM achieved high accuracy in predicting tourists′ preferences for visiting different destinations [25]. Additionally, studies on protected areas in Sarawak, Malaysia, have utilized various ML models, including k‐NN, naive Bayes, and decision trees, to classify visitor arrivals. Among these, the decision tree model demonstrated the best performance [30].

Sustainable tourism depends on human capital—the knowledge, skills, and motivation of individuals essential for delivering high‐quality services [29]. Easily accessible accommodations, food centers, shopping outlets, places of interest, events, and activities, as well as the ambience and accessibility in any tourism destination not only brings about satisfaction but also dissatisfaction to the tourists [31]. These observations showcase the relationship between service quality, marketing, and technological innovations with tourists, in particular, in the underdeveloped area like Bangladesh.

## 3. Materials and Methods

### 3.1. Research Design and Participants

The research focused on quantitative data only in this case, by the use of a structured questionnaire to determine the effect of the service marketing mix, popularly known as 7Ps—product, price, place, promotion, people, process, and physical evidence—on the tourists′ satisfaction in Bangladesh. The focus of the study was specifically to show that there is a linkage between the 7Ps and tourist satisfaction through quantitative and qualitative techniques as well as ML approaches.

The aim was to study the perceptions, preferences, and experience of the tourists in relation to these areas of the marketing mix. This was achieved through strategic focus groups which were meant to strengthen the validity of each question in regard to the wider dimensions of satisfaction attributed to each construct. The data gathering exercise was done at Cox′s Bazar, in Bangladesh, which is best known as the most extended natural sea beach in the world and a major center for local and foreign tourism. The ethical approval was obtained from Noakhali Science and Technology University Ethical Committee (NSTUEC), and all participants provided informed consent prior to completing the survey. The survey was anonymous, and participants were assured of confidentiality and voluntary participation.

The target population for this study includes tourists who travel to Cox′s Bazar, the seashore in Bangladesh. These respondents were particularly targeted as they could share their experiences and insights with regard to the service marketing mix (7Ps) and their corresponding satisfaction. The participants of the study were those who were consecutively and actively associating themselves in tourism activities.

### 3.2. Questionnaire Design

This study′s questionnaire was designed in a two‐tier structure to allow for deeper data collection. The first section contained questions aimed at obtaining sociodemographic data such as age, sex, education, income, and travel. These are the profiles of the demographic data that positioned the tourists and how they may affect the level of satisfaction.

The second section included 25 items aimed at assessing tourists′ perceptions toward the service marketing mix (7Ps), namely, product, price, place, promotion, people, process, and physical evidence, and their effect on satisfaction. Each of the 7Ps was measured using specific questions covering such aspects as that contained in the marketing mix.

**Figure 1:**
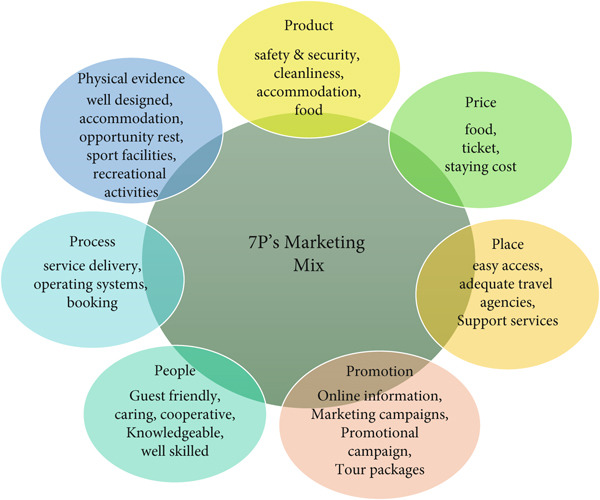
7P′s marketing mix.

The items were subsequently rated on a 5‐point Likert scale, in order to reveal the strength of the respondents′ feelings and experiences on the issues. This scale helped measure the level of satisfaction as well as the effect of marketing mix elements of the respondents which made available credible data for further analysis. 7P′s marketing mix elements are displayed in (Figure 1).

### 3.3. Sampling

This study employs a nonprobability sampling approach to collect data from tourists visiting Cox′s Bazar in Bangladesh. A sample size of 500 respondents was selected to ensure sufficient statistical power and reliability for analysis. In this study, nonprobability sampling was necessary because Cox′s Bazar is a busy and diverse tourist area with no complete list of visitors, and arrivals change by day, location, and season. As our survey was conducted during the peak tourist season, a full probability‐based design was not feasible. We collected data from multiple busy and distinct locations (Laboni/Kolatoli area, Marine Drive/Inani points, Himchari, and hotel clusters) using consistent inclusion and exclusion criteria. Our relatively large sample size (*n* = 500) improved representativeness, and we checked the stability of results through principal component analysis (PCA) and cross‐validation.

Participants were approached based on a systematic interval, such as every fifth individual who met the inclusion criteria. The inclusion criteria specified that participants had to be 18 years or older and actively engaged in tourism activities during the data collection period. Exclusion criteria included local residents and individuals not participating in tourism‐related activities, ensuring that the responses represented actual tourists.

Data collection was conducted by trained personnel stationed at multiple key locations within Cox′s Bazar. This strategic placement of data collectors ensured the diversity of the sample and captured a wide range of demographic characteristics and travel motivations, enhancing the generalizability of the findings. Efforts were made to minimize nonresponse bias by engaging tourists during peak activity times and diversifying collection sites.

### 3.4. Data Analysis

The reliability of the survey instrument was confirmed using Cronbach′s alpha, a method for assessing reliability. A variable is considered reliable if its Cronbach′s alpha value is above 0.7 [32]. Multicollinearity was assessed using the variance inflation factor (VIF). High VIF values and low tolerance values indicated the presence of multicollinearity, suggesting that variables were highly correlated and could distort the model′s reliability [33]. If these values are considerably high or low, it suggests significant multicollinearity that needs to be addressed. PCA was employed to further reduce dimensionality and manage any remaining multicollinearity. PCA is a statistical technique that transforms correlated variables into a smaller set of uncorrelated components, retaining most of the variance within the data [34]. Various visualization techniques were utilized to gain a deeper understanding of data distribution and relationships among factors, including histograms, violin plots, density plots, correlation heatmaps, and box plots. These visualizations provided a comprehensive overview of the data, helping identify key trends and outliers. Multiple statistical models were developed to predict tourist satisfaction based on the collected data. Both traditional regression techniques and advanced ML algorithms were employed, including linear regression, decision tree, random forest, gradient boosting, XGBoost, and support vector regression (SVR).

#### 3.4.1. Linear Regression

Linear regression is one of the simplest and most widely used regression models. It assumes a linear relationship between the independent variables (features) and the dependent variable (target) [35]. The model is aimed at minimizing the sum of squared differences between the observed and predicted values. The general form of the linear regression equation is

y=β0+β1x1+β2x2+⋯+βnxn+ϵ

where *y* is the predicted target variable, *x*_1_, *x*_2_ ⋯ *x*_*n*_ are the input features, *β*_0_ is the intercept, *β*_1_ *β*_2_ ⋯ *β*_*n*_ are the coefficients for each feature, and *ϵ* is the error term.

Linear regression uses the ordinary least squares (OLS) method to find the best‐fitting line by minimizing the sum of squared residuals:

Minimize=∑i=1nyi−y∧i2

where *y*_*i*_ is the actual value and ${\overset{\wedge }{y}}_{i}$ is the predicted value.

#### 3.4.2. Decision Tree

**Figure 2:**
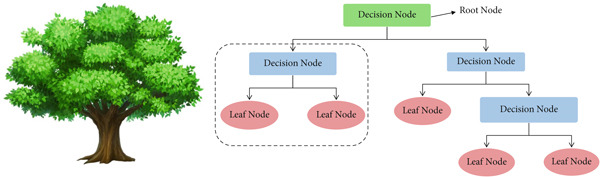
Decision tree.

A decision tree is a nonlinear model that splits the data into subsets based on feature values, creating a tree‐like structure of decisions (Figure 2). At each internal node, a decision rule is applied based on a feature, and branches are created based on the possible outcomes. The tree continues to split until a stopping criterion is met, such as a maximum tree depth or minimum samples per leaf [36]. The decision tree algorithm uses a criterion like mean squared error (MSE) for regression tasks to measure the impurity of each split. At each node, the algorithm chooses the feature and split that minimizes the MSE. The formula for the MSE at a node is

MSE=∑i=1nyi−y∧i2.

where *y*_*i*_ is the actual value and ${\overset{\wedge }{y}}_{i}$ is the predicted value at the node.

#### 3.4.3. Random Forest

**Figure 3:**
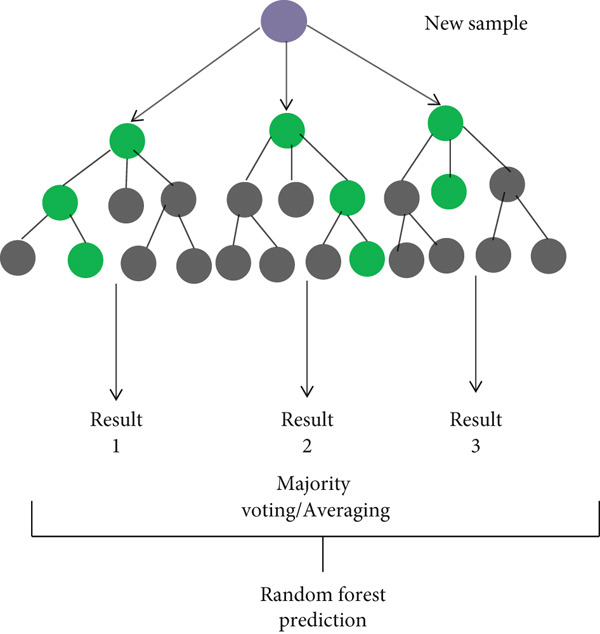
Random forest.

Random forest is an ensemble learning method that combines multiple decision trees to improve predictive performance. Random forest, developed by Leo Breiman and Adele Cutler, is a popular ML algorithm that combines multiple decision trees to produce a single output. The model creates multiple decision trees by randomly selecting subsets of the data and features. Each tree in the forest is trained independently, and predictions are made by averaging the predictions of all trees. In the bootstrap method used for random forest, *N* datasets are prepared by randomly sampling *N* records with replacement from the original data. This forms the foundation for building the trees. For a dataset with *M* input features, a smaller number of *m* (*m* ≪ *M*) is randomly selected at each node. The best split is determined from these *m* features and is used to divide the node. The value of *m*, often referred to as *m*_try_ or *k* in the literature, remains constant throughout the construction of the forest. Each tree is grown to its maximum depth, and the total number of trees *N*_tree_ is specified as a hyperparameter before training [37]. Figure 3 represents a random forest classifier taken from this source [38].

#### 3.4.4. Gradient Boosting

Gradient boosting is an ensemble technique that builds models sequentially, where each new model corrects the errors made by the previous one. It minimizes the residual errors by fitting new models on the residuals of the previous predictions. The gradient boosting technique trains an ensemble of *M* trees sequentially, where each tree corrects the errors of the previous one. The process begins with Tree 1, which is trained on the feature matrix *X* and the labels *y*, producing predictions. ${\overset{\wedge }{\;y}}_{i}$ is used to compute residual errors *r*_1_. Tree 2 is then trained on *X* and residual errors *r*_1_ as the target labels, and this process continues, with each tree addressing the residuals from its predecessor, forming a sequence of residuals. A critical aspect of this method is shrinkage, where each tree′s predictions are scaled by a learning rate (*η*), which ranges between 0 and 1. Lower values of *η* require more trees to achieve comparable performance, creating a trade‐off between the learning rate and the number of estimators. Once all *M* trees are trained, predictions for new data are made by aggregating the scaled contributions of all trees [39]. This approach, often used in boosting algorithms like gradient boosting, relies on residual correction and shrinkage to build a highly accurate predictive model. The formula for predicting the final prediction is

yprediction=y1+ηr1+ηr2+.⋯⋯+ηrn.

Gradient boosting performs well when the data has complex relationships as it iteratively focuses on the most difficult cases.

#### 3.4.5. XGBoost

XGBoost is an optimized version of gradient boosting that incorporates additional regularization terms to reduce overfitting and improve performance [40]. XGBoost is a decision tree–based optimization technique that leverages the gradient descent method to refine the model. It optimizes the loss function using gradient descent while incorporating regularization parameters to mitigate overfitting. The core principle of the XGBoost algorithm is to reduce the objective function effectively [41]. The formula for XGBoost′s objective function is

Lt=∑i=1nℓyi,y∧it−1+fixi+Ωft

where *ℓ* is the loss function, $\ell \left(y_{i},{{\overset{\wedge }{y}}_{i}}^{t-1}+f_{i}\left(x_{i}\right)\right)$ is the loss function for *i*‐th data point, and *Ω*(*f*_*t*_) is a regularization term for the model *f*_*t*_.

XGBoost is known for its speed and accuracy, often outperforming other ML models in competitive scenarios.

#### 3.4.6. SVR

SVR is based on the SVM algorithm, which is typically used for classification tasks. In regression, the goal of SVR is to find a hyperplane that best fits the data while allowing for some margin of error [42]. The function of SVR is *f*(*x*) = 〈*w*, *x*〉 + *b*.

The optimization problem minimizes the following objective:

minw,b,ϵi12w2+c∑i=1nϵi.

SVR is aimed at finding a function that predicts target values with minimal error while maintaining a margin of tolerance *ϵ*.

**Figure 4:**
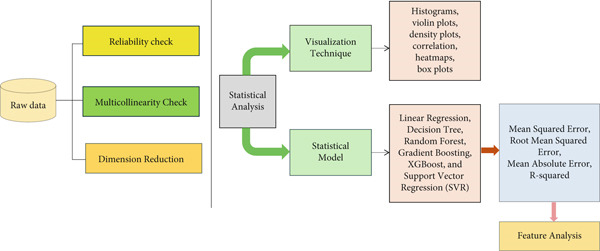
Study design flowchart.

Model performance was evaluated using metrics such as MSE, RMSE, MAE, and *R*‐squared values. Feature importance analysis was conducted to identify the most influential factors contributing to tourist satisfaction, ranking the predictors based on their impact on the model′s accuracy. To identify the most influential factors contributing to tourist satisfaction, a feature importance analysis was conducted. This analysis ranked the predictors based on their contribution to the model′s accuracy. The robustness of the selected model was confirmed through cross‐validation, a technique that splits the dataset into multiple folds to evaluate the model′s performance on different subsets of data [43]. This approach ensures that the model generalizes well to unseen data and is not overfitting to the training set. A complete design of this study is shown in the flowchart of Figure 4.

#### 3.4.7. Structural Equation Modeling (SEM)

In addition to the regression and ML models, SEM has also been incorporated as a traditional benchmark for examining the theoretical relationships among the latent constructs of the 7Ps and tourist satisfaction. SEM is widely used in tourism and service marketing research because it simultaneously assesses both the measurement model and the structural pathways linking constructs. Including SEM in our analysis provides a theoretical point of comparison to the predictive models such as linear regression, random forest, gradient boosting, and XGBoost. The SEM model was estimated using maximum likelihood, and its explanatory power (*R* ^2^ = 0.036) is reported alongside the predictive models in the Data Analysis and Results section.

## 4. Data Analysis and Results

The internal consistency of the constructs was evaluated using Cronbach′s alpha (Table 1), with all values exceeding the widely accepted threshold of 0.7, indicating satisfactory reliability of the survey instrument. Constructs such as satisfaction (0.88), price (0.86), and place (0.87) demonstrated high reliability, suggesting strong correlations among their items and a robust measurement of the intended dimensions. Moderately reliable constructs, including product (0.73), promotion (0.75), and people (0.73), showed acceptable reliability, though there is room for refinement to further enhance consistency.

**Table 1:** Reliability statistics.

**Construct**	**Cronbach**′**s alpha**
Satisfaction	0.88
Product	0.73
Price	0.86
Place	0.87
Promotion	0.75
People	0.73
Process	0.76
Physical evidence	0.80

Similarly, process (0.76) and physical evidence (0.80) displayed moderate‐to‐high reliability, affirming their alignment with the corresponding dimensions. While the overall reliability of the instrument is confirmed, constructs with relatively lower Cronbach′s alpha values may benefit from reviewing item–total correlations to identify and improve weaker items. These findings validate the survey′s reliability in measuring tourist satisfaction and the 7Ps of the service marketing mix.

To assess multicollinearity among predictors, VIF values were examined. Table 2 presents the VIF values for all predictors. The constant (const) shows an extremely high VIF of 487.11, which is expected and does not influence multicollinearity among predictors. However, price aggregated and place aggregated exhibit high VIF values of 12.11 and 12.09, respectively, exceeding the commonly accepted threshold of 10. These values suggest potential multicollinearity issues, indicating that these predictors may be highly correlated with others. Such correlations can distort the reliability of regression coefficients. Addressing this issue may involve removing or combining these variables or applying dimensionality reduction techniques like PCA.

**Table 2:** Variance inflation factor (VIF) values.

**Feature**	**VIF**
Price aggregated	12.11
Place aggregated	12.09
Product aggregated	1.00
Promotion aggregated	1.02
People aggregated	1.01
Process aggregated	1.01
Physical evidence aggregated	1.00

In contrast, other predictors—product aggregated, promotion aggregated, people aggregated, process aggregated, and physical evidence aggregated—have VIF values close to 1. This indicates no multicollinearity issues, confirming their independence and unique contribution to the model. Further investigation and remedial steps are necessary to resolve the multicollinearity observed with price aggregated and place aggregated, while the remaining predictors can confidently remain in the regression model.

After combining price and place into a single variable (price place aggregated), all VIF values are close to 1, as shown in Table 3. This indicates that the issue of multicollinearity present in the original dataset has been effectively resolved. The predictors now contribute independently and uniquely to the model, ensuring reliable regression coefficient estimates.

**Table 3:** Variance inflation factor (VIF) after combining variables.

**Feature**	**VIF**
Price place aggregated	1.02
Product aggregated	1.00
Promotion aggregated	1.03
People aggregated	1.01
Process aggregated	1.01
Physical evidence aggregated	1.00

The first principal component (PC1) explains 97.88% of the variance in the combined variable, while the second component (PC2) accounts for only 2.12% (Table 4). This indicates that the combined variable is predominantly influenced by a single underlying dimension, justifying the aggregation of price and place to reduce multicollinearity without significant information loss.

**Table 4:** Principal component analysis (PCA)–explained variance ratio.

**Principal component**	**Explained variance ratio**
PC1	97.88%
PC2	2.12%

Given these findings, the price place aggregated variable should be used in the model to simplify the analysis and address multicollinearity effectively. PCA confirms that this combination captures the majority of the variance, validating it as a robust approach for dimensionality reduction.

**Figure 5:**
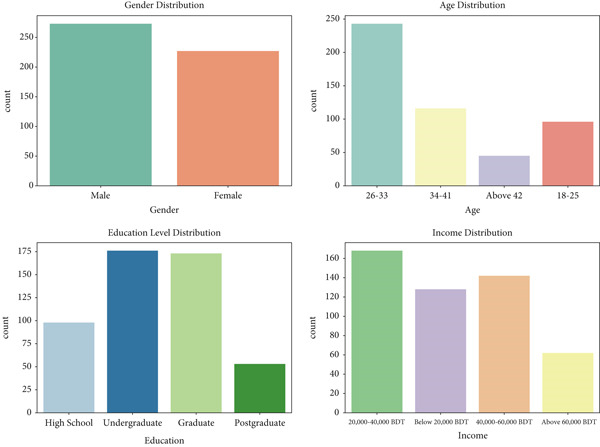
Distribution of various demographic variables.

The graphs (Figure 5) reveal that the dataset has a relatively balanced gender representation, with a slightly higher number of male respondents compared to females. In terms of age distribution, the largest group is 26–33 years, followed by 18–25 years, indicating that the dataset predominantly consists of younger individuals. Smaller proportions of respondents belong to the 34–41 years and above 42 years categories, further emphasizing the youthfulness of the sample.

Regarding education and income, most respondents have an undergraduate or graduate level of education, reflecting a highly educated population. High school and postgraduate categories are represented by fewer individuals. In terms of income, the majority fall within the 20,000–40,000 BDT range, followed by the 40,000–60,000 BDT range, while fewer respondents are in the below 20,000 BDT and above 60,000 BDT categories. Overall, the dataset is skewed toward younger, educated individuals with moderate income levels, which aligns with the likely target demographic of the study.

**Figure 6:**
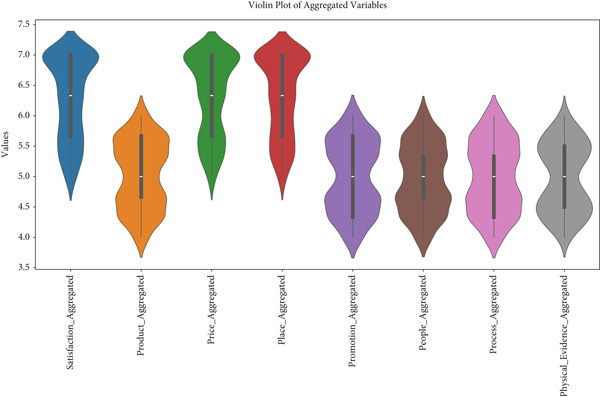
Violin plot (for distribution and density).

The violin plot (Figure 6) visualizes the distribution of values for various aggregated variables, highlighting the spread, density, and central tendencies within the dataset.

The variable satisfaction_aggregated demonstrates the highest concentration around a mean value near 7, with a relatively symmetrical distribution and a narrow spread, suggesting consistency among respondents. Product_aggregated, in contrast, shows a broader spread, with values ranging more widely, indicating greater variability in responses. Price_aggregated and place_aggregated have similar distributions, with central tendencies slightly below 6 and moderate spread, reflecting some variability but overall alignment in respondent perceptions.

Promotion_aggregated, people_aggregated, process_aggregated, and physical_evidence_aggregated all exhibit relatively symmetric distributions, with central tendencies around 5.5–6. These variables have moderate spreads, indicating consistent but slightly varied responses. Overall, the violin plot provides insights into the consistency and variability of the aggregated variables, suggesting that while some aspects, such as satisfaction, are highly consistent, others, like product and price, show more diverse responses.

The dataset comprises 500 observations for each aggregated variable, with consistent standard deviations (ranging from 0.558 to 0.679), indicating moderate variability within the data (Table 5). The variables satisfaction aggregated, price aggregated, and place aggregated exhibit the highest mean values (approximately 6.3), reflecting strong positive aggregation in these dimensions. These areas are likely well regarded by respondents.

**Table 5:** Summary statistics of aggregated variables.

**Metric**	**Count**	**Mean**	**Std dev**	**Min**	**25%**	**50%**	**75%**	**Max**
Satisfaction aggregated	500	6.29	0.68	5	5.67	6.33	7	7
Product aggregated	500	5.02	0.57	4	4.67	5	5.67	6
Price aggregated	500	6.31	0.66	5	5.67	6.33	7	7
Place aggregated	500	6.31	0.66	5	5.67	6.33	7	7
Promotion aggregated	500	4.99	0.59	4	4.33	5	5.67	6
People aggregated	500	4.96	0.55	4	4.67	5	5.33	6
Process aggregated	500	4.99	0.58	4	4.33	5	5.33	6
Physical evidence aggregated	500	4.96	0.567	4	4.5	5	5.5	6

In contrast, the variables promotion aggregated, people aggregated, process aggregated, and physical evidence aggregated show lower mean values (around 5), suggesting potential areas for improvement. The narrow range of values across all metrics, with a minimum of 4 and a maximum of 7, indicates a well‐constrained and uniformly distributed dataset (Table 5). These findings highlight the need for targeted enhancements in areas such as promotion strategies and people management to improve overall performance and perception.

The performance metrics (Table 6) clearly highlight XGBoost as the best‐performing model, achieving the lowest MSE (0.10), RMSE (0.33), and MAE (0.25), along with the highest *R*‐squared value (0.74). These results demonstrate that XGBoost explains the largest proportion of variance and provides the most accurate predictions among all models evaluated. Gradient boosting is a close second, with an *R*‐squared of 0.705987 and comparable error metrics, making it a highly competitive alternative for predictive tasks.

**Table 6:** Model performance.

**Model**	**MSE**	**RMSE**	**MAE**	**R** **-squared**
Linear regression	0.26	0.51	0.42	0.35
Decision tree	0.19	0.43	0.29	0.54
Random forest	0.12	0.35	0.28	0.67
Gradient boosting	0.12	0.34	0.27	0.70
XGBoost	0.10	0.33	0.25	0.74
Support vector regression (SVR)	0.28	0.53	0.43	0.39
SEM				0.036

Random forest ranks third, showing strong performance with relatively low error values and an *R*‐squared of 0.67, confirming its robustness in prediction. Decision tree, while moderately effective, has higher error metrics and a considerably lower *R*‐squared (0.54), indicating reduced predictive accuracy compared to ensemble methods. Linear regression and SVR exhibit the weakest performances, with the highest error rates and the lowest *R*‐squared values (0.35 for linear regression and 0.39 for SVR), suggesting limited ability to explain variance or make accurate predictions. Overall, XGBoost substantially outperforms traditional models, achieving far higher predictive accuracy than SEM, which shows a very low latent *R* ^2^ (0.0359) and poor fit indices (CFI = 0.2077, GFI = 0.3482, and TLI = −0.018).

**Figure 7:**
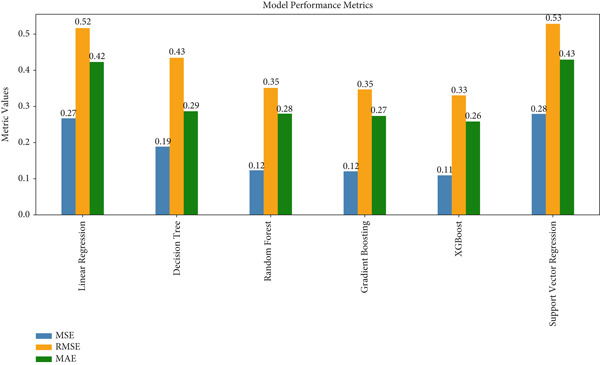
Bar plot of model performance metrics.

The bar plot in Figure 7 presents a comparative analysis of various ML models using three evaluation metrics: MSE, RMSE, and MAE. These metrics, which quantify prediction error, demonstrate that lower values correspond to superior model performance.

Among the models analyzed, XGBoost and SVR achieve consistently low error values across all three metrics, signifying their effectiveness and robustness in capturing the patterns of the dataset. Notably, their performance edges out that of other models, marking them as the most reliable predictors for this task.

The decision tree and random forest models also exhibit competitive performance, with moderate error values that suggest their capacity to model the data effectively, though they fall slightly short of the precision demonstrated by XGBoost and SVR. In contrast, linear regression and gradient boosting show comparatively higher error values, indicating that these models may be less suitable for this particular dataset.

**Figure 8:**
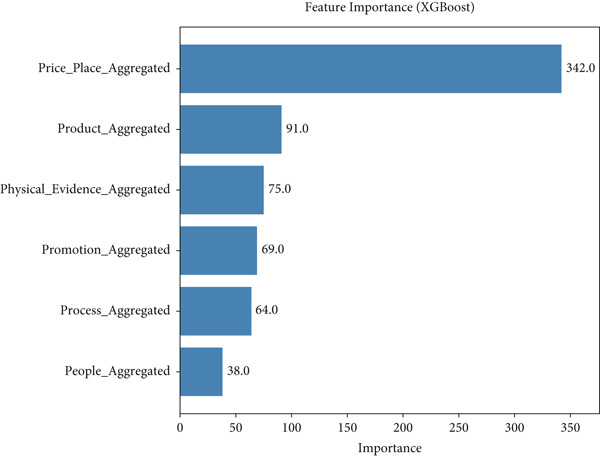
Feature importance of the XGBoost model.

The bar chart in Figure 8 highlights the contribution of individual features to the XGBoost model′s predictive performance, measured by the *F*‐score. This score quantifies how frequently a feature is used in the model′s decision‐making process, with higher values indicating greater significance. Among the analyzed features, “price place aggregated” emerges as the most influential, underscoring its pivotal role in shaping the model′s predictions.

Figure 8 displays the feature importance of a model. The *F*‐score represents the contribution of each feature to the model′s prediction. The higher the *F*‐score, the more important the feature. In this case, the “price place aggregated” feature is the most important, followed by “product aggregated” and “physical evidence aggregated.” This information can be used to understand the underlying factors driving the model′s predictions and potentially improve its performance.

The next most significant features are “product aggregated” and “physical evidence aggregated,” which also show strong *F*‐scores, reflecting their substantial impact on the model′s performance. These features collectively capture critical dimensions of the dataset, suggesting their importance in modeling the underlying relationships. Meanwhile, other features contribute less prominently, indicating their relatively lower influence on the model′s outcomes.

This analysis provides valuable insights into the factors driving the XGBoost model′s predictive power. By focusing on highly ranked features, stakeholders can better interpret the model′s behavior and potentially refine it further—either by enhancing the quality of key features or simplifying the model by excluding less impactful variables.

The cross‐validation results for the XGBoost model demonstrate its robustness and reliability, with a MSE of 0.1273 ± 0.0170. This indicates that the model consistently achieves low prediction errors across multiple validation folds, reflecting its ability to generalize well to unseen data.

The narrow margin of error (±0.0170) further highlights the stability of the model′s performance, suggesting that it is not overly sensitive to variations in the training dataset. This consistency strengthens confidence in the model′s predictive accuracy and underscores its suitability for making reliable forecasts on new data.

Overall, these cross‐validation results confirm that the XGBoost model is not only effective but also resilient, making it a strong candidate for deployment in scenarios where precise and dependable predictions are critical.

## 5. Discussions

The findings of this study offer significant insights into the predictive relationships between service marketing mix elements and tourist satisfaction, particularly in the context of Cox′s Bazar, Bangladesh. By utilizing ML techniques, this research not only validates existing theoretical frameworks but also introduces data‐driven methodologies for enhancing decision‐making in tourism management. The study highlights that the aggregated variable, price_place, exerts the most substantial influence on tourist satisfaction, followed by promotion and process. These results are consistent with prior studies emphasizing the importance of affordability, accessibility, and service quality in shaping tourists′ experiences [18, 44, 45].

The dominance of the price_place variable underscores the critical role of financial and logistical factors in destination choice and satisfaction. Competitive pricing strategies and convenient locations often serve as key differentiators for tourist destinations. This finding aligns with studies suggesting that price sensitivity and ease of access significantly impact tourists′ overall evaluations and likelihood of return visits [45, 46]. For Cox′s Bazar, policymakers can leverage this insight by implementing dynamic pricing strategies and improving transportation infrastructure to enhance accessibility, thereby boosting satisfaction and loyalty.

Promotion and process also emerged as significant contributors to satisfaction, which resonates with the broader literature on service quality and marketing effectiveness. Effective promotional campaigns, particularly those emphasizing the unique value propositions of Cox′s Bazar, can attract diverse tourist segments and improve satisfaction levels [47]. Similarly, efficient processes that minimize delays and enhance service reliability are critical for fostering positive tourist experiences. This is supported by research indicating that seamless service delivery enhances customer perceptions of reliability and trust, which are vital for satisfaction and loyalty [45, 48].

The study′s methodological rigor strengthens the reliability of its findings. With Cronbach′s alpha values exceeding 0.70 for all constructs, the internal consistency of the data is well within the acceptable range [18]. Additionally, the resolution of multicollinearity through variable aggregation and PCA ensures the accuracy of the predictive models. The ability of the first two principal components to explain 97.88% of the variance highlights the compactness and clarity of the dataset, reducing noise and enhancing interpretability. This methodological approach is a noteworthy contribution to tourism research, where data quality often influences the robustness of conclusions [49, 50].

Among the ML models employed, XGBoost demonstrated superior predictive performance, with an *R*‐squared value of 0.7332 and the lowest error metrics (MSE = 0.1093, RMSE = 0.3306). These metrics surpass those reported in similar studies applying ML to tourism satisfaction prediction, such as [18], who found *R*‐squared values ranging between 0.65 and 0.70 in comparable contexts. The robustness of XGBoost was further validated through cross‐validation, with consistent performance metrics across folds. This highlights its reliability for identifying key predictors of satisfaction, making it a valuable tool for practitioners and researchers alike.

The findings of this study hold strong relevance for other tourism‐dependent economies in the Global South, such as Sri Lanka, Vietnam, and Kenya, which similarly rely on natural attractions, cultural heritage, and service quality to drive visitor satisfaction and loyalty [2, 23, 31]. These destinations often face parallel challenges, including infrastructure limitations, the need for competitive pricing, and the importance of enhancing accessibility and promotional effectiveness [23, 51]. The prominence of factors such as affordability, location convenience, and streamlined service processes in our results aligns with priorities in these countries, where attracting repeat visitors and diversifying tourist markets are crucial for economic resilience [2, 44, 52]. By adopting the same ML‐based analytical framework, stakeholders in these contexts can uncover their own critical satisfaction drivers, enabling more targeted policy interventions and sustainable tourism growth [18, 25, 50].

The practical implications of these findings are profound. Tourism stakeholders in Cox′s Bazar can use these insights to prioritize investments in pricing strategies, infrastructure, and promotional campaigns. For example, dynamic pricing models that cater to different tourist segments can enhance affordability, while improved transportation networks can address accessibility concerns. In tandem, targeted promotional efforts that highlight the unique natural and cultural offerings of Cox′s Bazar can attract a broader audience, complementing improvements in service processes. Such integrated strategies are crucial for achieving sustainable tourism growth, as evidenced by studies emphasizing holistic marketing approaches [50].

The present study bridges the gap between theoretical constructs and practical applications in tourism satisfaction research. By integrating advanced statistical and ML techniques, it provides a nuanced understanding of the service marketing mix′s impact on satisfaction. While the findings are specific to Cox′s Bazar, they offer broader implications for other emerging destinations seeking to optimize their marketing strategies and enhance tourist experiences.

## 6. Conclusion and Recommendations

This study provides a comprehensive analysis of the predictive relationships between service marketing mix elements and tourist satisfaction, using advanced ML techniques to extract actionable insights. Focusing on Cox′s Bazar, Bangladesh, the research highlights the critical role of pricing strategies, location accessibility, promotional effectiveness, and service processes in shaping tourist satisfaction and loyalty. Among these, the aggregated price_place variable emerged as the most influential predictor, underscoring the importance of affordability and accessibility in the tourism industry.

The use of XGBoost as the primary predictive model demonstrated superior performance, with high accuracy and robust validation metrics, reinforcing its applicability in tourism research. The methodological rigor, including the resolution of multicollinearity, PCA‐based dimensionality reduction, and cross‐validation, ensures the reliability of the findings. These results advance the understanding of how service marketing mix elements interact to influence tourist perceptions and behaviors, providing both theoretical and practical contributions to the field.

For policymakers and tourism stakeholders in Cox′s Bazar, the findings offer strategic guidance for optimizing marketing efforts. Prioritizing pricing strategies that cater to diverse tourist segments, improving infrastructure to enhance accessibility, and designing targeted promotional campaigns can significantly enhance tourist experiences and foster loyalty. Additionally, streamlining service delivery processes will further strengthen the destination′s competitiveness. The key recommendations are to focus on tourism marketing that appeals to price‐conscious visitors, improve infrastructure to make locations more attractive, and create promotional strategies tailored to different groups of tourists. These suggestions include practical steps that local tourism authorities and stakeholders can follow to boost the sector.

While the study is geographically specific to Cox′s Bazar, the methodological framework and insights have broader implications for similar destinations worldwide. By adopting a data‐driven approach, this research demonstrates how ML can effectively identify and prioritize key drivers of tourist satisfaction, enabling stakeholders to make informed, impactful decisions.

Future research can build on these findings by exploring cross‐cultural variations, integrating real‐time data for dynamic modeling, and examining additional factors such as environmental sustainability and cultural heritage. Such endeavors will further refine predictive models and contribute to the development of sustainable tourism strategies.

## Conflicts of Interest

The authors declare no conflicts of interest.

## Author Contributions

Md. Nazmul Hoque: supervision, conceptualization of the idea, data collection, data preprocessing, software, writing of the manuscript, carrying out of manuscript editing and checking, and review of the manuscript. Sumiya Nur Jannat: data preprocessing, formal analysis, writing and review of the manuscript. Yasin Arafat: formal analysis and writing of the manuscript. Md. Mamun Miah: supervision, writing of the manuscript, carrying out of manuscript editing and checking, and review of the manuscript.

## Funding

No funding was received for this manuscript.

## Data Availability

Data and code are publicly available at https://github.com/STAT-Mamun/Cox-s-Bazar-Tourist-Satisfaction-data.
